# Picosecond pulse shaping of single photons using quantum dots

**DOI:** 10.1038/s41467-017-02552-7

**Published:** 2018-01-09

**Authors:** B. C. Pursley, S. G. Carter, M. K. Yakes, A. S. Bracker, D. Gammon

**Affiliations:** 1 NRC Research Associate residing at the Naval Research Laboratory, Washington, DC 20375 USA; 2 Naval Research Laboratory, Washington, DC 20375 USA

## Abstract

Quantum dots (QDs) are an excellent single-photon source that can be combined with a spin quantum memory. Many quantum technologies require increased control over the characteristics of emitted photons. A powerful approach is to trigger coherent Raman photons from QDs with a Λ energy-level system, such as the spin singlet–triplet system in two coupled QDs. The temporal and spectral behavior of single Raman photons can be varied simply by modifying the excitation source. Here, we demonstrate control of the single-photon pulse shape in a solid-state system on a timescale much shorter than the radiative lifetime, in addition to control of the frequency and bandwidth. We achieve a photon pulse width of 80 ps—an order of magnitude shorter than the exciton lifetime. Possible applications include time-bin encoding of quantum information, matching photons from different sources, and efficient single-photon transfer in a quantum network.

## Abstract (web summary)

Tailoring single photons’ pulse shapes could have applications such as time-bin encoding or interfacing different photon emitters. Here, the authors achieve pulse shaping of Raman single photons from InGaAs quantum dots on a picosecond timescale, much shorter than the radiative lifetime.

## Introduction

Indium arsenide quantum dots (QD) are one of the best sources of single photons available due to their bright, high-purity emission^1–3^. In addition, by injecting a single electron or hole, they can also serve as quantum spin memories. They have been embedded in a variety of electronic and photonic heterostructures in order to control the charge state^4,5^, enhance the emission rate^6–8^, increase the collection efficiency^9–11^, and obtain a spin–photon interface^12–17^. For many applications in quantum technology, there is a need to carefully tailor the properties of emitted photons. For example, photons from different quantum systems must be matched both spectrally and temporally^18^ in order for two-photon measurement-induced entanglement to occur. Moreover, the more difficult but much more efficient approach of using a deterministic protocol for entanglement and teleportation of quantum information between nodes in a quantum network also requires that the emitted photons be temporally symmetric^19,20^. The emission and absorption processes must be reversible so that a photon can be transferred to a stationary qubit without loss.

One powerful approach to address this challenge is to use coherent spin-flip Raman emission in a Λ energy-level system^21–25^, in which the drive laser and spontaneous emission coherently take the system from one spin state to another. In this process, the Raman photon properties are determined by the laser and by the spin properties, i.e., energy separation and coherence. Previous work has demonstrated that this technique can be used to tune the emitted photons up to ∼0.5 meV in photonic crystal cavities for both a single QD^16^ and a quantum dot molecule (QDM)^26^ and that the photons are highly indistinguishable^27^. We also expect that the temporal behavior of the photons from spin-flip Raman emission in QDs can be controlled. For detuned Raman emission, the temporal profile of the photon should follow that of the laser pulse, allowing for photons with arbitrary temporal profiles. In contrast, for two-level systems, the properties of the emitted photons are typically determined by the properties of the QDs. Recent results have also shown that coherent Rayleigh scattering from QD two-level systems can result in highly coherent emission determined by the properties of the laser, including control of the temporal profile^28^. However, this technique has so far been restricted primarily to resonant scattering limited by the QD linewidth, with emission times at least as long as the spontaneous emission time. Detuned Raman spin-flip emission has the advantages of a large bandwidth, spectral separation between the emitted photon and the laser pulse, and a ground-state spin system for quantum memory. This technique has been used with trapped ions and atoms in optical cavities to control single-photon wavepackets on a timescale of 0.1–1 μs^29,30^.

Here, we demonstrate pulse shaping of single photons down to the picosecond timescale, varying the emission time over an order of magnitude and producing temporally symmetric photons. We confirm single-photon emission and achieve Raman photon pulses of 80-ps duration—an order of magnitude shorter than the exciton lifetime.

## Results

### Energy-level system of a doubly charged QD molecule

**Fig. 1:**
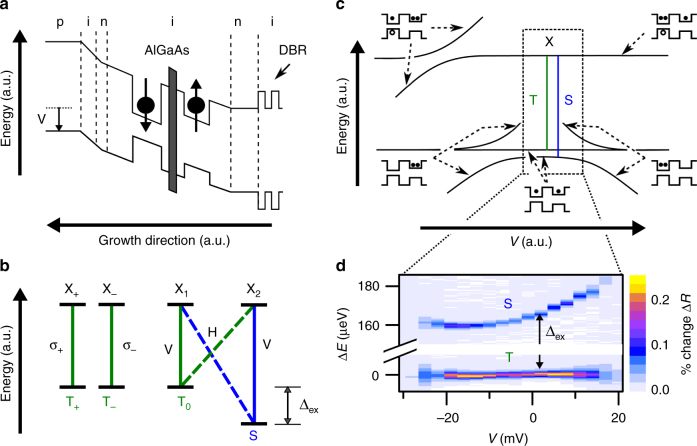
QDM two-electron charge configuration. **a** Schematic diode band structure and **b** energy-level diagram of the QDM two-electron charge configuration where one electron resides in each quantum dot. The electrons are tunnel coupled through a thin barrier (details in Methods) forming spin singlet (S) and triplet (T_0_, T_+_, and T_−_) ground states. The S and T_0_ states form two overlapping Λ systems with two exciton states through vertical (V, solid) and horizontal (H, dashed) linearly polarized optical transitions. **c** Schematic bias dependence of the lowest ground and excited energy levels for a QDM charged with two electrons. When both electrons reside in one quantum dot, they form a spin singlet and anti-cross with S, pushing the energy level down by the exchange splitting (∆_ex_). The boxed region corresponds to the bias range where two electrons are stable in the QDM, determined by **d** differential reflectance spectra. All energies are given relative to the triplet transition energy (1307.8 meV) and labeled as ∆*E*

The sample consists of two InGaAs QDs separated by a thin vertical tunnel barrier and embedded in a diode heterostructure (Fig. 1a)^31,32^. Biasing the diode allows us to tune the charge configuration of the QDM such that one electron is stable in each QD (Supplementary Fig. 1). Coherent tunneling of the two electrons between the two QDs leads to an exchange splitting (∆_ex_ ~ 160 µeV) between the spin singlet (S) and the three degenerate triplets (T_0_, T_+_, and T_−_). Optical transitions from the S and T_0_ states to the exciton states form two overlapping Λ systems with selection rules shown in Fig. 1b. Transitions of the T_+_ and T_−_ are circularly polarized and do not couple directly to the Λ systems. The S–T_0_ Λ system is similar to a single electron or hole in a single QD in a transverse magnetic field but has some potential advantages. The exchange splitting in the ground state of the QDM can be varied over several orders of magnitude and exists even at zero magnetic field^33,34^. In addition, as a spin memory, the QDM system is less sensitive to fluctuating electric fields or nuclear spins^35^.

Figure 1c displays the energies of the ground states and excited states as a function of bias, with the region where there is one electron in each QD outlined with a rectangle. The optical transitions of the singlet and triplet are displayed as a function of bias and laser detuning in the differential reflectivity^5^ map of Fig. 1d, showing the tuning of the transitions and the bias range where the two-electron configuration is stable. The intensity of the signal decreases by 50% at the center of this stability range due to weak optical pumping, in which the laser excites one spin state and drives the population to the other spin state^22,34,36^. The sample structure is designed (Methods) such that fast spin relaxation from tunneling to the n-doped region prevents complete optical pumping, providing a convenient way to reset the Raman spin-flip cycle and continue to emit photons.

### Spin-flip Raman emission

**Fig. 2:**
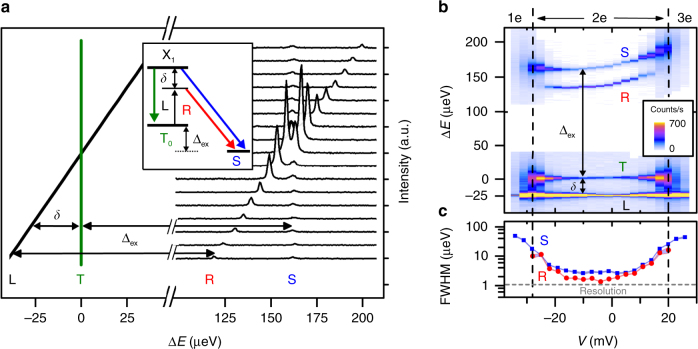
Spin-flip Raman in a QDM. **a** Emission spectrum of the QDM at a bias of −10 mV for a series of laser detunings, stepping across the triplet transition. The laser (L) and triplet (T) transition energies are indicated on the left, centered at ∆*E *= 0, where ∆*E* is the emission energy minus the triplet transition energy (1307.8 meV). The inset energy-level diagram illustrates the Raman (R), singlet PL (S), and triplet PL (T). **b** Emission spectrum colormap as a function of bias and ∆*E*, with *δ *= − 25 µeV. **c** Linewidth of R and S as a function of bias

Spin-flip Raman is a two-photon inelastic scattering process involving a transition between the spin singlet and triplet ground states. The Raman emission has linear polarization opposite to that of the drive laser (see Fig. 1b and the inset to Fig. 2a), which allows polarization rejection of scattered laser light while retaining all of the Raman emission. Figure 2a shows the emission spectrum for a fixed bias (−10 mV) as the laser frequency is tuned through the triplet resonance. There are two emission lines, both of which involve a spin-flip transition of the QDM, and both of which are enhanced in intensity on resonance. One is shifted from the laser by the exchange splitting (∆_ex_) and tracks the laser frequency. This line, labeled “R” for Raman, only involves a virtual transition to the exciton state and is not dephased by exciton dynamics. The other line, labeled “S” for singlet, is fixed at the singlet transition frequency and involves the excitation of a real population in the exciton state, followed by emission, i.e., quasi-resonant photoluminescence (PL). In what follows, we compare the properties of these two lines, first under continuous-wave (cw) excitation and then under pulsed excitation

In Fig. 2b, c, we fix the laser detuning 25 µeV below the triplet transition (*δ *=− 25 µeV) and scan the bias through the two-electron charge stability region. Near the stability edges, the linewidth of both the Raman and PL is very broad. This can be explained by relaxation of the ground-state spin through rapid cotunneling to the heavily doped electron layer^37^. In the middle of the stability region where cotunneling is weaker, both linewidths are an order of magnitude narrower. The PL linewidth has a minimum of ~3 µeV. Throughout the middle of the stability region, the Raman linewidth is ~1.7 µeV, which is 57% of the PL linewidth. This is an indication that the Raman emission linewidth is not necessarily determined by the transition linewidth and can instead be determined by the ground-state spin dynamics and the laser properties^38^. The Raman linewidth is still much larger than the laser linewidth (∼neV) due to various effects including spin relaxation and nuclear spin interactions. As we show next, when the laser linewidth is made larger than the Raman linewidth using pulsed excitation, the Raman emission reflects the laser linewidth and is not limited by the ground-state dynamics, in contrast to the PL, which remains the same.

### Pulse-shaping Raman photons

**Fig. 3:**
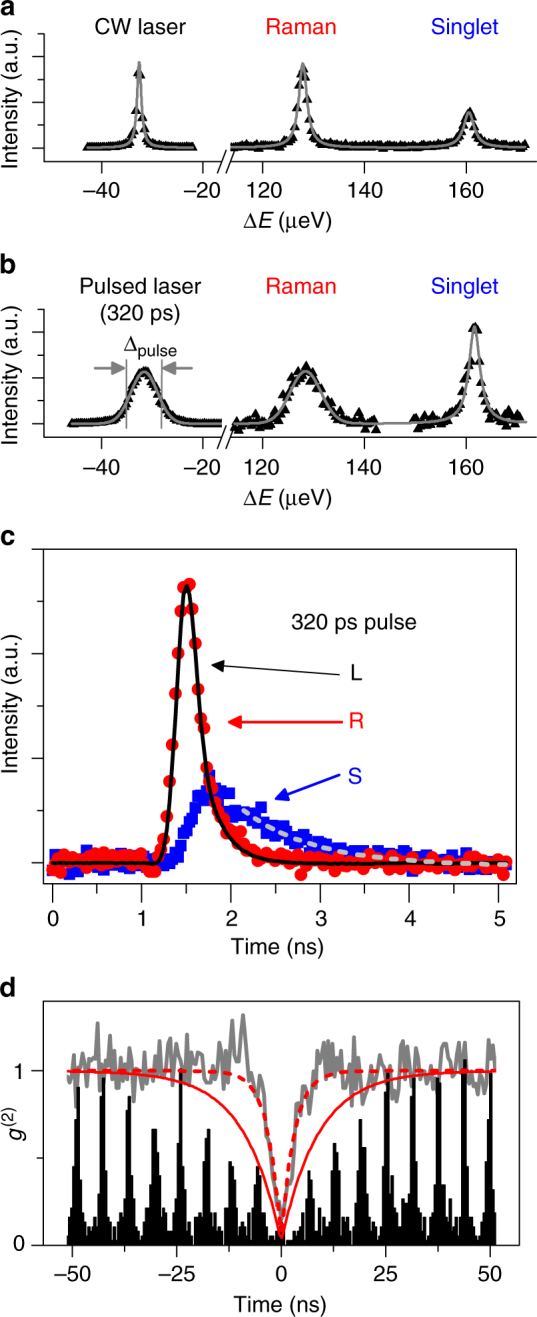
Pulse-shaping spin-flip Raman. **a**, **b** Spectrum of the laser, Raman emission, and singlet PL for **a** the cw laser and **b** a 320-ps pulsed laser, with *δ *= − 35 µeV. **c** Temporal profile of the laser (L), the Raman (R), and the singlet (S) emission with *δ *= − 50 µeV. **d** The second-order correlation function for pulsed (black line) and cw (gray line) excitation under similar conditions with fits (solid and dashed red lines, respectively). For cw excitation, the rise time was 3 ns; for pulsed, the rise time was 9 ns

The coherence of the Raman process allows properties of the laser, such as pulse shape as well as detuning, to be mapped onto the emission. In Fig. 3a, b we show the spectra at fixed bias (−10 mV) and detuning (*δ = *35 μeV) with cw excitation, and also with a 320-ps Gaussian pulse. Under cw excitation (Fig. 3a), the laser linewidth is narrower than the measured Raman and PL linewidths. For the 320-ps laser pulse (Fig. 3b), the laser’s spectral linewidth (∆_pulse_ = 6 µeV) is mapped onto the Raman emission, broadening the emission to approximately three times the cw linewidth. The PL linewidth remains unchanged as expected because the incoherent nature of PL erases information about the excitation source.

In Fig. 3c, the corresponding temporal shapes of the laser, the Raman, and the PL are plotted. The intensity of the singlet PL decays exponentially with a lifetime of 0.8 ns due to spontaneous emission. In contrast, the Raman intensity exactly tracks that of the laser. For pulses resonant with the triplet (not shown), the emission intensity does not follow that of the laser pulse and instead matches the PL temporal profile. Resonant Raman emission is more complicated because the PL and Raman emission are at the same energy and the sharp spectral response should modify the temporal behavior of pulsed Raman.

We verified that the detuned Raman emission consists of no more than one photon at a time by obtaining the second-order correlation function *g*^(2)^(*τ*) (Fig. 3d) for both pulsed and cw excitation. The two-photon probability *g*^(2)^(0) is ∼ 0.13 for both cases. The rise time away from *τ* = 0 represents how quickly a second Raman photon can be emitted. We expect that this rise time is determined by spin relaxation in the case of this Λ system. Since the rise time is slower for pulsed excitation (9 ns) than for cw (3 ns), we suspect that continuous interaction of the cw laser with the QD can more rapidly reset the spin system. Aside from cotunneling-induced spin relaxation, optical processes such as Stokes Raman or detuned absorption of the singlet transition can reset the spin system. These processes can occur continuously for a cw laser but are limited by the pulse repetition rate for pulsed excitation.

**Fig. 4:**
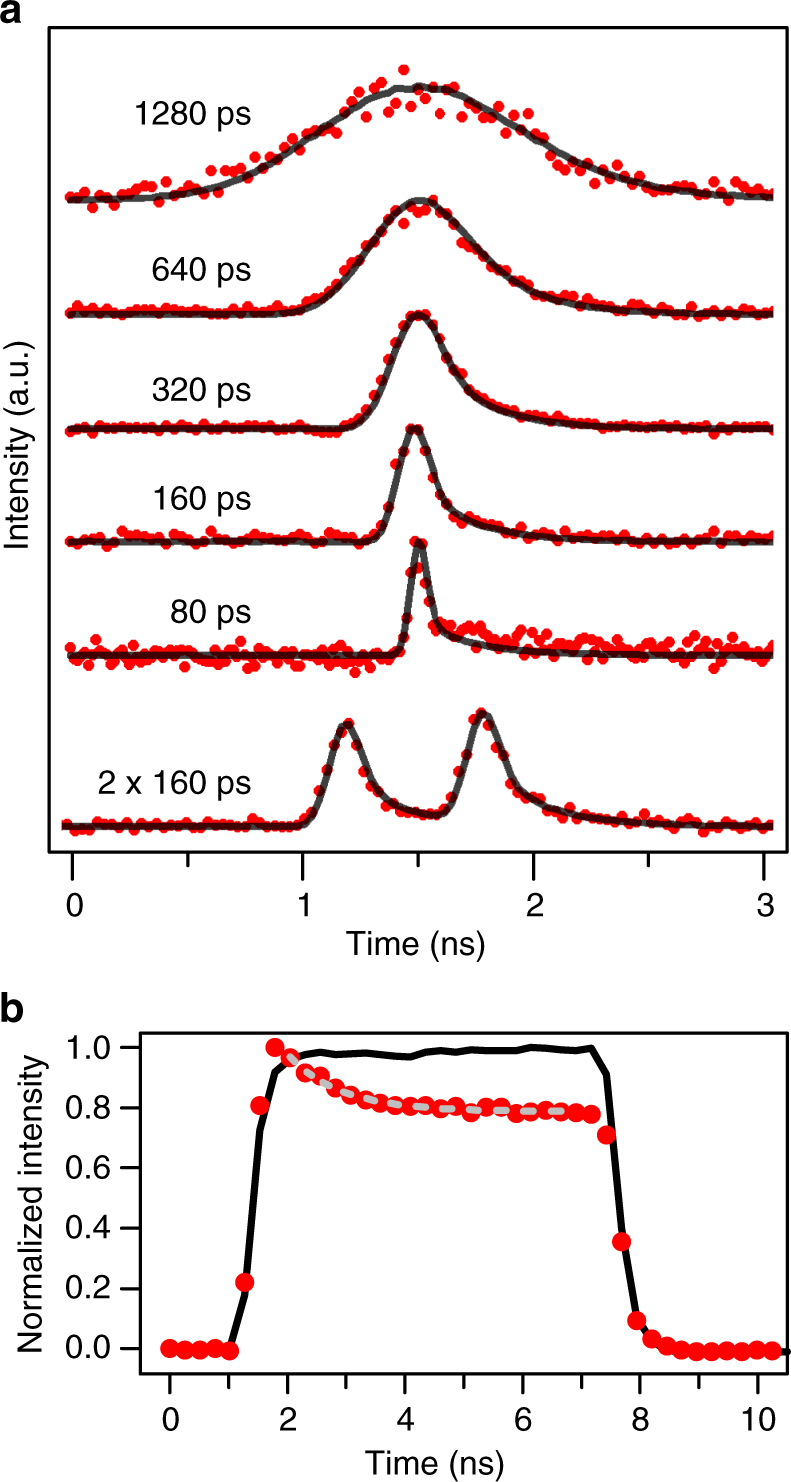
Temporal tuning. Temporal profile of the Raman emission at **a** *δ *= − 50 µeV for a series of laser pulse widths and a double pulse; **b** *δ *= −35 µeV with a 6-ns square-wave pulse. Solid lines are the laser intensity and the red circles are the Raman intensity. All laser pulses in **a** are symmetric Gaussian pulses. The asymmetry in the measured spectra arises from the response of the detector. The smaller detuning in **b** and a laser power of 1 µW were chosen to bring the equilibrium emission near saturation where the spin relaxation time should be the limiting factor (Supplementary Note 1). A value of 2.3 ns for the spin relaxation time was extracted from a fit (dashed gray line), in agreement with the rise time of the second-order correlation function measured with cw excitation (Fig. 3)

In Fig. 4a, we demonstrate that the temporal profile of the Raman emission tracks that of the laser for pulses of over 1-ns width down to 80 ps, which is the limit of our electronic pulse generator. This is an order of magnitude faster than spontaneous emission. The emission is temporally symmetric with any asymmetry in the data due to the finite response of the detector. In addition, we also demonstrate in the bottom trace of Fig. 4a that more complex lineshapes are possible with an example of two 160-ps pulses separated by 600 ps. For pulse separation less than the spin coherence time, we expect that this intensity profile still represents the wavepacket of a single-photon split between two time bins^39^.

## Discussion

How short temporally, or broad spectrally, we can make the Raman pulse will be limited by detuning and the magnitude of ∆_ex_. The linewidth of the laser pulse ∆_pulse_ must be less than *δ* in order to avoid the complications of resonant excitation. In addition, ∆_pulse_ should be less than ∆_ex_ so that reverse spin-flip transitions are unlikely and to avoid spectral overlap between the laser pulse and the Raman. We can engineer ∆_ex_ over multiple orders of magnitude, but larger values of ∆_ex_ typically result in shorter spin relaxation times^26,34,35,40^. For the conditions of Fig. 4a with *δ *= −50 μeV, the pulses should be longer than 40 ps to avoid these limits. With larger *δ* and ∆_ex_, the Raman pulses could be made even shorter.

The other limit of how long temporally, or narrow spectrally, we can make the Raman pulse, while retaining a single photon, will be determined by a combination of the spin relaxation time and the Raman spin-flip transition rate. In Fig. 4b, the temporal profile of Raman emission is displayed for a long 6-ns square pulse, in which a clear decay is observed. For negligible spin relaxation, the emission should decay to zero with the Raman spin transition rate because only one photon can be emitted until the spin is reset. With significant spin relaxation over this timescale, another photon can be emitted, so that there is little decay before reaching a steady-state intensity, which will obviously contain multiple photons. By modeling the data in Fig. 4b, we obtain a spin relaxation time of ∼2.3 ns under these conditions (Supplementary Note 1), which is consistent with the rise time of *g*^(2)^(*τ*) under cw excitation. For the shorter pulses in Fig. 4a, there is a small but significant probability of spin relaxation. This may explain, at least in part, the value of *g*^(2)^(0). The spin relaxation time can be increased by enlarging the tunnel barrier to the n-doped region or by decreasing ∆_ex_. For this study, it was convenient to limit the spin relaxation time to a value comparable to the spontaneous emission time in order to defeat optical pumping and effectively reset the Raman emission process. For some applications, a long spin memory is required in which case the spin state would need to be reset optically^27,41^.

The brightness of the Raman emission is also of great importance. For detuned Raman emission, there are three factors that can affect the efficiency. First, there is the collection efficiency, which is an issue common to all QD emission. Second, there is the Raman process efficiency, which is reduced by competing processes that can occur, such as PL or detuned Rayleigh scattering (Supplementary Fig. 2). Third, there is the initialization fidelity into the proper spin state before each pulse. As discussed in detail in Supplementary Note 2, the first two factors can be substantially improved using microcavities with Purcell enhancement, and the last factor can be improved with fast spin initialization. Currently, the brightness of detuned Raman emission is low, with approximately 10^−4^ photons emitted into the collection objective per pulse for 80-MHz excitation with 320-ps pulses. This gives a detector count rate of hundreds of counts/s. However, through the improvements mentioned above and in Supplementary Note 2, the brightness of Raman emission could be as high as with other optical excitation techniques^9–11^.

We have demonstrated that the temporal profile of photons emitted from QDs can be controlled over a wide range of timescales, from over 1 ns to 80 ps or less, using Raman spin-flip emission with a QDM. The same approach could also be taken with spin-flip Raman on an electron or hole spin in a single QD. This technique can be used to match the temporal profile of emitters with naturally different lifetimes and also allows for simple generation of time-bin-encoded photons^42^. The ability to arbitrarily control the photon wavepacket is particularly important for producing time-symmetric photons essential for deterministic transfer of quantum states^19^, which may allow for efficient scaling up of the number of qubits in a quantum network. Combined with spectral tuning of photons, this technique will be a valuable tool for applications in quantum photonic technologies.

## Methods

### Sample structure

Two vertically stacked InGaAs self-assembled dots were separated by a tunnel barrier composed of GaAs/Al_0.3_Ga_0.7_As/GaAs (3 nm/3 nm/3 nm) using molecular beam epitaxy (Fig. 1). The spin relaxation rate (and the amount of optical pumping) increases with the exchange splitting and also depends on the spacer layer thickness between the n-type layer and the QDs. By adjusting the relative thickness of the Al_0.3_Ga_0.7_As layer in the barrier in a series of samples, we optimized the spin exchange energy and spin relaxation rate for this study. For this sample with ∆_ex_ ~ 160 μeV, we found that the spin relaxation rate was large enough to avoid significant optical pumping but small enough to give narrow Raman linewidths^26,34,35,40^. Using the partial cap and In-flush technique, the dots were truncated to nominal heights of 1.9 nm and 2.8 nm^43^. The larger thickness of the second dot allows for alignment of the electron energy levels at a field where one electron is stable in each dot. The QDs were embedded in a diode heterostructure and grown on top of a distributed Bragg reflector (DBR) and an n-type GaAs substrate. The DBR provides improved collection of emission, while the diode allows for deterministic control of the charge configuration of the dots. The Si-doped DBR consists of ten periods of AlAs/GaAs, 82-nm and 69-nm thick, respectively. The diode structure is n–i–n–i–p (91 nm/114.7 nm/10 nm/21 nm/40 nm) with the dots located 40 nm into the first intrinsic layer. Silicon was used for the n-type dopant, beryllium for the p-type. The purpose of the second n-type layer is to reduce the current through the device at the bias where one electron is stable in each QD.

### Measurement methods

The sample was mounted in a ceramic chip carrier and placed in a closed-cycle He cryostat at a temperature of 4 K. An objective lens was used to achieve a 1-µm spot size for excitation. In all experiments, the incident laser light was vertically polarized. For differential reflectivity (Fig. 1d), the sample bias was modulated with a square wave at 317 Hz with an amplitude of 100 mV, while a cw laser was tuned through the resonances. Reflected laser light passed through a nonpolarizing beam splitter and was sent through a single-mode fiber to an avalanche photodiode. The generated voltage signal was demodulated using a lock-in amplifier with a 300-ms time constant.

For Raman spectra measurements, the emission was filtered through a horizontal linear polarizer followed by a scanning Fabry–Perot interferometer (FPI) locked to a tunable reference laser. The resolution and free spectral range of the FPI are 1 μeV and 440 μeV, respectively. The reference laser was ~50 meV below the QD emission energy, with its frequency monitored by a precision wavemeter, and a short-pass filter prevented the reference laser from getting mixed in with QD emission. As the reference laser was scanned, the FPI maintained its lock, providing a calibrated scan of the interferometer. The filtered emission was sent to a spectrometer for detection on a CCD with a 1-s (Fig. 2a), 4-s (Fig. 2b and Fig. 3a), or 16-s (Fig. 3b) exposure time. In Fig. 2, spectra were taken as a function of bias and detuning using 200 nW. In Fig. 3a, b, the sample was held at −10-mV bias, −35-µeV detuning, and 200 nW (cw) or 30 nW (320-ps pulse) of incident laser power.

Pulsed measurements of the QD emission were performed with either a mode-locked Ti:Sapphire laser (TSL) producing ∼300-ps pulses at a repetition rate of ∼81 MHz or with a cw diode laser passed through a fiber-coupled electro-optic modulator with >10-GHz bandwidth. An arbitrary waveform generator with a sampling rate of 50 GSamples/s was used to produce electronic pulses to drive the modulator, producing pulses as short as 80 ps. The spectral measurement in Fig. 3b was taken with the mode-locked laser since leakthrough of the cw diode laser through the modulator produced sharp features in the spectra. Time-resolved measurements were performed with the modulated cw laser, with emission only going through the spectrometer with ∼70-μeV resolution in order to avoid the strong temporal broadening of the FPI. The emission was resolved in time using a silicon photon-counting module with 50-ps timing response and also a longer ∼200-ps tail that gives an asymmetric temporal response. This fast detector has a few percent quantum efficiency at 950 nm and was used with a time-correlated single-photon counting module with 4-ps resolution. The second-order correlation functions *g*^(2)^(*τ*) in Fig. 3d were measured with a Hanbury Brown–Twiss interferometer and two Si photon-counting modules with higher quantum efficiency (∼23%) and lower timing resolution (∼300 ps). The average cw laser power was 10 µW. For pulsed laser measurements of *g*^(2)^(*τ*), the repetition rate of the TSL was doubled to ~162 MHz with an external optical delay line and an average laser power of 300 nW.

### Data availability

The data that support the findings of this study are available from the corresponding author upon reasonable request.

## Electronic supplementary material

Supplementary Information

## References

[1] Buckley S, Rivoire K, Vučković J (2012). Engineered quantum dot single-photon sources. Rep. Prog. Phys..

[2] Gazzano O, Solomon GS (2016). Toward optical quantum information processing with quantum dots coupled to microstructures. J. Opt. Soc. Am. B.

[3] Aharonovich I, Englund D, Toth M (2016). Solid-state single-photon emitters. Nat. Photonics.

[4] Warburton R (2000). Optical emission from a charge-tunable quantum ring. Nature.

[5] Alén B, Bickel F, Karrai K, Warburton RJ, Petroff PM (2003). Stark-shift modulation absorption spectroscopy of single quantum dots. Appl. Phys. Lett..

[6] Yoshie T (2004). Vacuum Rabi splitting with a single quantum dot in a photonic crystal nanocavity. Nature.

[7] Englund D (2005). Controlling the spontaneous emission rate of single quantum dots in a two-dimensional photonic crystal. Phys. Rev. Lett..

[8] Noda S, Fujita M, Asano T (2007). Spontaneous-emission control by photonic crystals and nanocavities. Nat. Photonics.

[9] Nowak AK (2014). Deterministic and electrically tunable bright single-photon source. Nat. Commun..

[10] Somaschi N (2016). Near-optimal single-photon sources in the solid state. Nat. Photonics.

[11] Ding X (2016). On-demand single photons with high extraction efficiency and near-unity indistinguishability from a resonantly driven quantum dot in a micropillar. Phys. Rev. Lett..

[12] Gao WB, Fallahi P, Togan E, Imamoglu A (2012). Observation of entanglement between a quantum dot spin and a single photon. Nature.

[13] Greve KDe (2012). Quantum-dot spin-photon entanglement via frequency downconversion to telecom wavelength. Nature.

[14] Schaibley JR (2013). Demonstration of quantum entanglement between a single electron spin confined to an InAs quantum dot and a photon. Phys. Rev. Lett..

[15] Carter SG (2013). Quantum control of a spin qubit coupled to a photonic crystal cavity. Nat. Photonics.

[16] Sweeney TM (2014). Cavity-stimulated Raman emission from a single quantum dot spin. Nat. Photonics.

[17] Sun S, Kim H, Solomon GS, Waks E (2016). A quantum phase switch between a single solid-state spin and a photon. Nat. Nanotechnol..

[18] Meyer HM (2015). Direct photonic coupling of a semiconductor quantum dot and a trapped ion. Phys. Rev. Lett..

[19] Cirac J, Zoller P, Kimble H, Mabuchi H (1997). Quantum state transfer and entanglement distribution among distant nodes in a quantum network. Phys. Rev. Lett..

[20] Liu RB, Yao W, Sham LJ (2010). Quantum computing by optical control of electron spins. Adv. Phys..

[21] Kiraz A, Atatüre M, Imamoğlu A (2004). Quantum-dot single-photon sources: prospects for applications in linear optics quantum-information processing. Phys. Rev. A.

[22] Atatüre M (2006). Quantum-dot spin-state preparation with near-unity fidelity. Science.

[23] Santori C, Fattal D, Fu KMC, Barclay PE, Beausoleil RG (2009). On the indistinguishability of Raman photons. New J. Phys..

[24] Fernandez G, Volz T, Desbuquois R, Badolato A, Imamoglu A (2009). Optically tunable spontaneous Raman fluorescence from a single self-assembled InGaAs quantum dot. Phys. Rev. Lett..

[25] Sipahigil A (2016). An integrated diamond nanophotonics platform for quantum-optical networks. Science.

[26] Vora PM (2015). Spin–cavity interactions between a quantum dot molecule and a photonic crystal cavity. Nat. Commun..

[27] He Y (2013). Indistinguishable tunable single photons emitted by spin-flip Raman transitions in InGaAs quantum dots. Phys. Rev. Lett..

[28] Matthiesen C (2013). Phase-locked indistinguishable photons with synthesized waveforms from a solid-state source. Nat. Commun..

[29] Keller M, Lange B, Hayasaka K, Lange W, Walther H (2004). Continuous generation of single photons with controlled waveform in an ion-trap cavity system. Nature.

[30] Nisbet-Jones PBR, Dilley J, Ljunggren D, Kuhn A (2011). Highly efficient source for indistinguishable single photons of controlled shape. New J. Phys..

[31] Stinaff EA (2006). Optical signatures of coupled quantum dots. Science.

[32] Krenner H (2006). Optically probing spin and charge interactions in a tunable artificial molecule. Phys. Rev. Lett..

[33] Doty M (2008). Optical spectra of doubly charged quantum dot molecules in electric and magnetic fields. Phys. Rev. B.

[34] Kim D, Carter SG, Greilich A, Bracker AS, Gammon D (2011). Ultrafast optical control of entanglement between two quantum-dot spins. Nat. Phys..

[35] Weiss KM, Elzerman JM, Delley YL, Miguel-Sanchez J, Imamoğlu A (2012). Coherent two-electron spin qubits in an optically active pair of coupled InGaAs quantum dots. Phys. Rev. Lett..

[36] Xu X (2007). Fast spin state initialization in a singly charged InAs-GaAs quantum dot by optical cooling. Phys. Rev. Lett..

[37] Dreiser J (2008). Optical investigations of quantum dot spin dynamics as a function of external electric and magnetic fields. Phys. Rev. B.

[38] Sun Z, Delteil A, Faelt S, Imamoglu A (2016). Measurement of spin coherence using Raman scattering. Phys. Rev. B.

[39] Lee, J. P. et al. Multi-dimensional photonic states from a quantum dot. Preprint at http://arxiv.org/abs/1703.10403 (2017).

[40] Elzerman JM, Weiss KM, Miguel-Sanchez J, Imamoǧlu A (2011). Optical amplification using Raman transitions between spin-singlet and spin-triplet states of a pair of coupled In-GaAs quantum dots. Phys. Rev. Lett..

[41] Delteil A (2016). Generation of heralded entanglement between distant hole spins. Nat. Phys..

[42] Brendel J, Gisin N, Tittel W, Zbinden H (1999). Pulsed energy-time entangled twin-photon source for quantum communication. Phys. Rev. Lett..

[43] Bracker AS (2006). Engineering electron and hole tunneling with asymmetric InAs quantum dot molecules. Appl. Phys. Lett..

