# Supplementary material for: Picosecond pulse shaping of single photons using quantum dots
Source: Nat Commun. 2018 Jan 9;9:115. doi: 10.1038/s41467-017-02552-7 (PMC5760648; doi:10.1038/s41467-017-02552-7)
Supplement: Supplementary file 1 — Supplementary Information [file 41467_2017_2552_MOESM1_ESM.pdf]

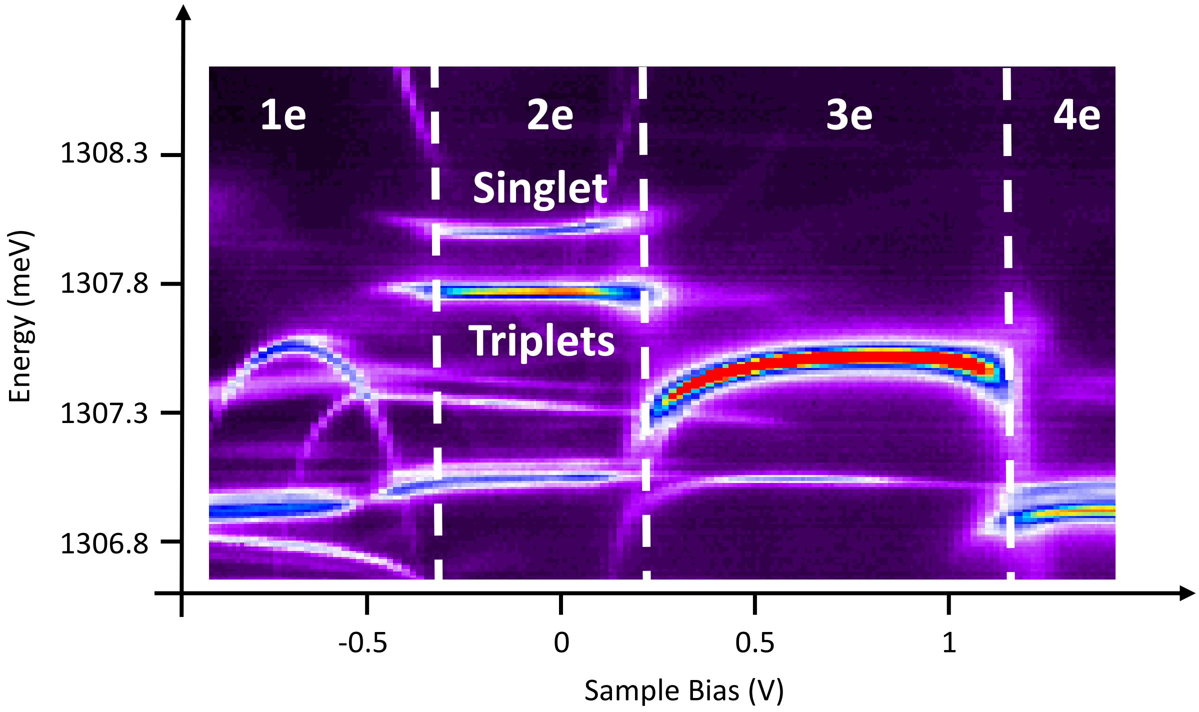

Supplementary Figure 1 | Bias Dependent Photoluminescence. Nonresonant PL spectra were measured as a function of voltage bias with laser excitation at 1370 meV and 10 uW of power. A one second exposure using a triple spectrometer operating in additive mode with a CCD camera provided resolution of 25 μeV. The voltage bias range over which the two-electron (2e) charge configuration of the QDM is stable is determined roughly by this PL spectral-bias map followed by the higher-resolution reflectance spectral map shown in Fig. 1.

Supplementary Note 1. Spin relaxation model

The spin-flip Raman process only virtually requires an excited state, so we use a simple two-level rate equation model to determine the spin relaxation rate between the spin singlet and triplet ground states. For our experiment of near-resonant Raman excitation from the T_0 to the S state (α) and spin relaxation (β) between the two states, we have

$$\dot{\rho_1} = -(\alpha+\beta)\,\rho_1 + \beta\,\rho_2 \qquad (1)$$
$$\dot{\rho_2} = (\alpha+\beta)\,\rho_1 - \beta\,\rho_2 \qquad (2)$$

where ρ_1 is the population in T_0, ρ_2 is that in S. Assuming that at time $t = 0$, $\rho_1 = \rho_2$ and that $\rho_1 + \rho_2 = const$, we obtain

$$\rho_1(t) \propto\ 2\beta + (\alpha)\exp[-(\alpha+2\beta)t] \qquad (3)$$

We justify our first assumption in that the energy splitting of the ground states (~160 µeV) is less than $k_B T$ (~330 µeV at 3.8 K) and our second from fact that T_+ and T_- do not couple directly to the Λ-system.

The spin relaxation rate (β) is obtained from the data in Figure 4b using the ratio of the transient amplitude to the steady state value in the data ($r = \alpha/2\beta \sim 0.27$) and the intensity decay rate ($\gamma = \alpha + 2\beta \sim$ 1 GHz). Solving yields, $\beta = \gamma/2(r+1)$. We obtain β = 0.4 GHz and $\alpha = 0.2$ GHz. This value of spin relaxation obtained from Fig. 4b is consistent with a value of ~3 ns (0.3 GHz) extracted from the rise time in the cw second order correlation data (inset Fig. 3 c).

Supplementary Note 2. Optimizing brightness of Raman single photon emission

The brightness of single photon generation is critical for practical quantum information applications. In the current experiments, the efficiency has not been optimized. There are three main areas where the brightness of Raman emission using InAs QDs can be optimized: collection efficiency, Raman process efficiency, and spin initialization fidelity.

Collection efficiency from InAs QDs and QDMs can be improved by orders of magnitude through the use of a cavity or waveguide, which can very efficiently couple photons into a single optical mode. Recent demonstrations involving InAs QDs have yielded collection efficiencies as high as 80%[1] into free space and 98% into a photonic crystal waveguide[2]. This challenge is the same faced by all QD emitters, although for spin-flip Raman, the emission polarization is perpendicular to that of the laser excitation. This is valuable in laser suppression, especially in combination with a linearly polarized cavity or waveguide.

The Raman process efficiency is determined by the probability of driving the Raman process and by competition with other processes, in particular incoherent PL emission and Rayleigh scattering. Specific emission pathways can be enhanced through the use of cavity-induced Purcell enhancement of a particular transition. Supplementary Fig. 2 displays all of the possible processes for the case of the horizontally polarized (H) laser detuned from the S state (dashed blue line), with a cavity tuned to the Stokes Raman photon (solid green line). We consider a simple picture of the different optical processes and how they can be modified to increase efficiency. The Raman process results in a vertically polarized (V) photon detuned below the triplet transitions. Unlike Rayleigh scattering, cross polarization eliminates scattered laser light without reducing the collection efficiency. Rayleigh scattering of the laser back to the S state (dashed red line) is also possible, which we expect to occur with about equal probability as the Raman process since the transition dipoles for X_1 to S and T_0 are roughly equal.

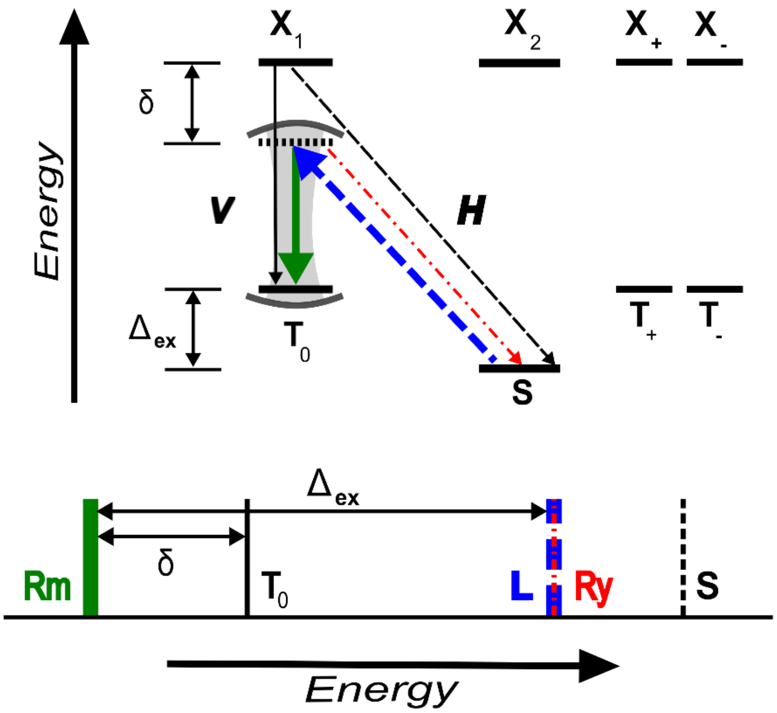

Supplementary Figure 2 | QDM 2e Energy Levels and Associated Spectra . Top: The energy level diagram of a two-electron QDM. The system would be initialized into the singlet (S) state and a horizontally polarized laser pulse detuned form the singlet transition would excite the cavity-stimulated Raman transition into the T_0 state with vertical polarization. Also shown are competing transitions consisting of Rayleigh scattering back to the singlet state as well as the two PL transitions. Δ_{ex} is the S-T_0 exchange splitting, δ is the laser detuning. H and V are horizontal and vertical polarizations respectively. Bottom: A schematic of the emitted energies from the top energy level diagram. Rm is the Raman emission, T_0 is the incoherent emission between X_1 and T_0, S is the incoherent emission between X_1 and S, L is the laser scatter not involving the QDM, and Ry is Rayleigh scatter from the QDM.

Incoherent PL emission at the S or T_0 transition (dashed and solid black lines) can also compete with Raman emission when a real exciton population is excited, followed by spontaneous emission. This can occur in the presence of dephasing[3] or through phonon-assisted absorption. In Fig 2 of the main text, with the laser near the T_0 transition we observe clear PL emission throughout the bias map, but it is stronger near the edges of the charge stability region where spin relaxation is rapid. We postulate that rapid spin relaxation increases the proportion of incoherent PL emission. Indeed, we find in another study that Raman spectra from a single QD sample charged with a single hole showed negligible PL emission, presumably because of longer spin relaxation times. Reducing

spin relaxation for the QD molecule can be accomplished by enlarging the tunnel barrier to the n-doped region or by decreasing Δ_{ex}.

We can express the relative efficiency of Raman emission as

$$\epsilon = \frac{\Gamma_{Rm}}{\Gamma_{Rm} + \Gamma_{Ry} + \Gamma_{abs}}, \tag{4}$$

where Γ_{Rm} and Γ_{Ry} are the Raman and Rayleigh scattering rates and Γ_{abs} is the the sum of incoherent PL due to absorption. To estimate ϵ, we assign the relative values of these rates to the emission intensities from a spectrum of Fig. 2(b) and assume $\Gamma_{Rm} = \Gamma_{Ry}$. With a ratio of Raman to PL emission of 0.74, ϵ is estimated at 0.3. For a linearly polarized cavity mode aligned with the Raman photon and orthogonal to the laser, there should be Purcell enhancement of the Raman process and not Rayleigh scattering. Absorption processes giving rise to incoherent emission should also not be enhanced. For a Purcell factor of 10, obtained in a photonic crystal cavity[4], only Γ_{Rm} is multiplied by 10, giving a relative efficiency of $\epsilon = 0.81$. With slower spin relaxation, this efficiency could be even higher. However, these values are based on data taken where the overall excitation probability is still much less than one. As the pulse intensity is increased to improve the absolute efficiency, the states should be significantly dressed by the laser field, the competing processes may pose more of a challenge, and dephasing induced by the strong field may occur. The use of a cavity, however, may reduce the required laser power and limit these effects. Further work is needed to determine the process efficiency under these conditions. We are encouraged, however, by results in atomic systems where the efficiency of the Raman process can be 66%[5].

In the current work, the initial spin state could be in any of the four spin states with roughly equal probability since $k_B T$ is more than double the exchange splitting Δ_{ex} and there is fast spin relaxation. The highest efficiency would be obtained by initializing into the S state, which is isolated and lower in energy than T_0 (see Supplementary Figure 2). The initialization can be accomplished thermally, provided $\Delta_{ex} \gg k_B T$, which can occur in QDMs with higher Δ_{ex}[6]. A better approach, which does not rely on fast spin relaxation, is to optically initialize into the S state with a laser pulse tuned to the T_0 transition. This has been accomplished in a QDM with a fidelity of at least 95%[7] and should occur on a few ns timescale. We therefore expect that these improvements could result in near 100% initialization efficiency.

Overall, assuming 80% collection efficiency, 81% Raman process efficiency, and 95% initialization fidelity, the total brightness could be 62% per cycle, comparable to state of the art QD sources but with the ability to tune the spectral and temporal profile of the photons.

Supplementary References

1. Gazzano, O. & Solomon, G. S. Toward optical quantum information processing with quantum dots coupled to microstructures. *J. Opt. Soc. Am. B* **33,** C160–C175 (2016).
2. Arcari, M. *et al.* Near-Unity Coupling Efficiency of a Quantum Emitter to a Photonic Crystal Waveguide. *Phys. Rev. Lett.* **113,** 093603 (2014).
3. Mukamel, S. *Principles of Nonlinear Optical Spectroscopy*. (Oxford University Press, 1999).
4. Toishi, M., Englund, D., Faraon, A. & Vuckovic, J. High-brightness single photon source from a quantum dot in a directional-emission nanocavity. *Opt. Express* **17,** 14618–14626 (2009).
5. Nisbet-Jones, P. B. R., Dilley, J., Ljunggren, D. & Kuhn, A. Highly efficient source for indistinguishable single photons of controlled shape. *New J. Phys.* **13,** 103036 (2011).
6. Vora, P. M. *et al.* Spin–cavity interactions between a quantum dot molecule and a photonic crystal cavity. *Nat. Commun.* **6**:7665 doi: 10.1038/ncomms8665 (2015).
7. Kim, D., Carter, S. G., Greilich, A., Bracker, A. S. & Gammon, D. Ultrafast optical control of entanglement between two quantum-dot spins. *Nat. Phys.* **7,** 223–229 (2011).
